# Phenylalanine hydroxylase mRNA rescues the phenylketonuria phenotype in mice

**DOI:** 10.3389/fbioe.2022.993298

**Published:** 2022-10-07

**Authors:** Maximiliano L. Cacicedo, Christine Weinl-Tenbruck, Daniel Frank, Maria Jose Limeres, Sebastian Wirsching, Katja Hilbert, Mansure Abdollah Pasha Famian, Nigel Horscroft, Julia B. Hennermann, Fred Zepp, Frédéric Chevessier-Tünnesen, Stephan Gehring

**Affiliations:** ^1^ Children’s Hospital, University Medical Center of the Johannes-Gutenberg University, Mainz, Germany; ^2^ CureVac AG, Tübingen, Germany; ^3^ Atriva Therapeutics GmbH, Tübingen, Germany

**Keywords:** phenylketonuria (PKU), phenylalanine hydroxylase (pah), metabolic diseases, mRNA-based therapy, sapropterin

## Abstract

Phenylketonuria (PKU) is an inborn error of metabolism caused by a deficiency in functional phenylalanine hydroxylase (PAH), resulting in accumulation of phenylalanine (Phe) in patients’ blood and organs. Affected patients encounter severe developmental delay, neurological deficits, and behavioral abnormalities when not treated. Early diagnosis and treatment are extremely important; newborn screening programs have been implemented in most countries to ensure early identification of patients with PKU. Despite available treatment options, several challenges remain: life-long adherence to a strict diet, approval of some medications for adults only, and lack of response to these therapies in a subpopulation of patients. Therefore, there is an urgent need for treatment alternatives. An mRNA-based approach tested in PKU mice showed a fast reduction in the accumulation of Phe in serum, liver and brain, the most significant organ affected. Repeated injections of LNP-formulated mouse PAH mRNA rescued PKU mice from the disease phenotype for a prolonged period of time. An mRNA-based approach could improve the quality of life tremendously in PKU patients of all ages by replacing standard-of-care treatments.

## Introduction

Phenylketonuria (PKU) is an autosomal recessive inborn error of metabolism caused by a deficiency of the hepatic enzyme phenylalanine hydroxylase (PAH). PAH catalyzes the degradation of phenylalanine (Phe) to tyrosine (Tyr) in the presence of the co-factor, tetrahydrobiopterin (BH_4_). PAH dysfunction leads to the accumulation of Phe in blood and organs, generating disease-related pathologic symptoms. Untreated patients suffer from severe neurological impairment evidenced by intellectual disability, behavioral, movement, and psychiatric problems (van Spronsen et al., 2021). Even though PAH is abundant in liver tissue, the liver is not affected in PKU patients (Blau et al., 2010). Phe, whose levels are elevated in the serum of patients with PKU, crosses the blood-brain barrier and accumulates in the brain, causing detrimental effects on brain development and function (Grisch-Chan et al., 2019; Pilotto et al., 2021). The incidence of PKU is variable, depending upon population demographics and geographic zone; the incidence, for example, ranges from 1:10,000 in Caucasian populations to 1:2,700 in Italy (Hillert et al., 2020). Early diagnosis and treatment are extremely important; consequently, most countries conduct screening of newborns to guarantee early identification of affected patients (Grisch-Chan et al., 2019). A life-long Phe-restricted diet is fundamental to disease management (Azen et al., 1991; van Spronsen et al., 2021). Often, however, dietary treatment is not entirely effective even though it might have been initiated during the first days after birth. Even PKU patients under optimal treatment have demonstrated some neuropsychological problems and lower IQ scores compared with matched healthy individuals. The severity of these effects is dependent on the adherence to diet (Arnold et al., 2004; van Spronsen et al., 2021). Long-term complications might include osteopenia (mostly likely due to deficiencies in micronutrients resulting from dietary treatment), osteoporosis (slight increase in PKU patients has been observed), and chronic kidney disease (Hennermann et al., 2013; de Castro et al., 2020). Pregnant women are an especially vulnerable patient cohort; high levels of Phe are neurotoxic to the developing fetus, leading to developmental delay, microcephaly, and congenital heart disease (Grisch-Chan et al., 2019).

Currently, there are two approved drugs on the market used to treat phenylketonuria. Sapropterin dihydrochloride (marketed as Kuvan^®^) is a BH_4_ co-factor analog, which improves phenylalanine tolerance. Sapropterin dihydrochloride therapy requires daily oral administration and is effective in treating only 20%–30% of PKU patients who suffer from mild PKU with residual PAH activity; often, these patients must still adhere to a diet low in Phe (Keil et al., 2013; Muntau et al., 2017; van Wegberg et al., 2021). The pegylated form of phenylalanine ammonia lyase (PEG-PAL), a non-mammalian enzyme that degrades Phe *via* a pathway different than PAH, offers a second treatment option. PEG-PAL administered daily *via* subcutaneous injections can ease dietary restrictions in many cases though it is approved for adults only, due to potential severe side effects (Gupta et al., 2018; Sacharow et al., 2020).

To date, efforts to find an improved treatment for PKU include alternate therapeutic strategies that address PAH dysfunction (Grisch-Chan et al., 2019; Regier et al., 2022). Most of these approaches use viral vectors to deliver a DNA construct containing the sequence that encodes PAH. Adeno-associated viruses (AAVs) are most commonly used as delivery vehicles. It has been reported, that delivery of a functional codon-optimized human *Pah* gene generates PAH activity in a PKU mouse model (Ahmed et al., 2020; Kaiser et al., 2021). This approach can experience a loss of efficacy over time, due to the proliferation of the infected cells. Moreover, immune responses to the viral vector limits repeated administrations (Hamilton and Wright, 2021). Gene editing tools such as CRISPR/Cas9 offer a means of permanently correcting mutated *Pah*. Indeed, studies conducted in PKU-deficient mice showed a sustained reduction in Phe levels (Richards et al., 2020; Böck et al., 2022). Despite promising results, however, it remains unclear whether this approach can reach clinical application in light of possible off-target effects.

Alternatively, mRNA-based therapy affords a powerful tool with tremendous potential to treat a variety of indications, e.g., infectious disease, personalized cancer therapy, protein replacement and gene editing (An et al., 2017; Apgar et al., 2018; Roseman et al., 2018; Hauser et al., 2019; Kowalski et al., 2019; Zhu et al., 2019; Jiang et al., 2020; Kowalzik et al., 2021; Wei et al., 2021). Formulated in lipid nanoparticles (LNPs), mRNA encoding the therapeutic protein (PAH in this study) is transported *via* the blood stream to the liver where it is taken up and expressed primarily by hepatocytes. LNP-mediated delivery of small interfering RNA (siRNA) to the liver was already approved by the FDA (Onpattro^®^ (patisiran) as a therapeutic option (Adams et al., 2018; Akinc et al., 2019). Moreover, LNP-formulated mRNA vaccines injected intramuscularly were used worldwide to fight the coronavirus pandemic over the past few years (Corbett et al., 2020; Polack et al., 2020; Sahin et al., 2020; Gebre et al., 2022; Kremsner et al., 2022).

Among the different PKU models described in the literature, two mouse models have been extensively used for the study of novel PKU therapies. The PKU phenotype in both models was generated by random ethylnitrosourea-induced mutagenesis. The resulting *Pah* ^*enu1*^ mutation represents a model of mild hyperphenylalaninemia, whereas mice expressing the *Pah* ^*enu2*^ mutation exhibit a severe PKU phenotype (Shedlovsky et al., 1993). The severe PKU mouse model was used in a study by Perez-Garcia et al. describing an mRNA-based therapeutic approach for PKU. Therapeutic efficacy was shown for LNP-formulated mRNA encoding for human PAH (Perez-Garcia et al., 2022). In the present study, intravenous (IV) injection of mouse *Pah* (*MmPah*) mRNA formulated in LNPs targeted production of therapeutic PAH protein to the livers of *Pah* ^*enu2*^ (PKU) mice. Single and repeated IV injections of *MmPah* mRNA-LNPs reduced pathologic increases of Phe in serum, liver and, importantly, in brain. Taken together, these findings suggest that LNP-formulated *MmPah* mRNA could provide an alternate treatment option for PKU patients. This approach would benefit patients of all ages by preventing the adverse complications and difficulties associated with standard-of-care treatment, i.e., life-long adherence to a Phe-restricted diet.

## Materials and methods

### Ethics statement

Ethical approval of the protocol titled: “Analysis of phenylalanine content in blood from phenylalanine hydroxylase deficient mice by targeted nanoparticle-mediated incorporation of phenylalanine hydroxylase-encoding mRNAs into hepatocytes” was granted by the Landesuntersuchungsamt LUA, Koblenz, AK G 18-1-086 on 5 December 2018. This approval is valid until 31 December 2023. Mice were provided unlimited access to food and water. All animal procedures were conducted according to the regulations of local authorities (Landesuntersuchungsamt Rhineland-Palatinate, G 18-1-086).

### Cell culture

HepG2 (ATCC HB-8065) and BHK-21 (ATCC CCL-10) cells, purchased from the American Type Culture Collection (Manassas, VA, United States), were grown in RPMI (Gibco BRL) supplemented with 10% FBS, 2 mM glutamine, 100 μg/ml streptomycin and 100 U/mL penicillin. After 24 h incubation, cells were transfected using Lipofectamine MessengerMax according to the protocol provided by the manufacturer (ThermoFisher, Plainville, MA, United States). Briefly, cells were seeded into 12-well tissue culture plates and transfected with 1 μg *MmPah* mRNA using 2 μl Lipofectamine. *EGFP* mRNA was used as a positive transfection control in each experiment. To collect PAH for enzymatic activity assessment, 100 μl RIPA buffer (ThermoFisher) with Halt™ Protease Inhibitor Cocktail (ThermoFisher) were added to each well. Cell lysates were collected and stored at −80°C for further analysis.

### Phenylalanine hydroxylase enzymatic assay

PAH produced after transfection was collected by lysing the cells with RIPA lysis buffer (ThermoFisher) and incubating for 30 s at room temperature. Fresh lysates were placed on ice until use. For enzyme analysis, 200 μl of cell lysate were incubated for 5 min at room temperature with 80 μl of HEPES buffer (30 mM), 35 μl bovine liver catalase (20 mg/ml; Sigma-Aldrich, St. Louis, MO, United States) and 35 μl L-phenylalanine (10 mM; Sigma-Aldrich). Then, 70 μl ammonium iron (II) sulfate hexahydrate (100 μM; Sigma-Aldrich) and 250 μl HEPES (30 mM) were added and incubated for 1 min at room temperature. Finally, 26.4 μl of tetrahydro-L-Biopterin (BH_4_) (5 mg/ml; Cayman Chemical Company, Ann Arbor, MI, United States) dissolved in dithiothreitol (2 mM; Applichem Inc., Council Bluffs, IA, United States) were added to the reaction mixture, and the mixture was incubated for 3 h at 30°C with agitation (300 rpm). Samples were collected and stored at −80°C for further analysis. Final concentrations of reagents in the reaction mix were: 1 mg/ml catalase, 0.5 mM L-Phe, 10 μM ammonium iron (II) sulfate hexahydrate and 600 μM BH_4_.

### *MmPah* mRNA-LNP formulation

An mRNA sequence encoding murine PAH (*MmPah* mRNA) was designed and synthesized *in vitro*. The mRNA construct was GC-optimized to improve translation and half-life of the mRNA. The mRNA contains a 5′ UTR from the human hydroxysteroid 17- beta dehydrogenase 4 gene (HSD17B4), an open-reading frame (ORF) encoding mouse PAH, a 3′ UTR from human proteasome 20S subunit beta 3 gene (PSMB3), and a template-encoded poly(A) sequence. mRNAs were enzymatically capped to obtain Cap1 and enzymatically polyadenylated. mRNAs were generated using non-modified nucleotides.

*MmPah* mRNA was formulated in LNPs for *in vivo* injection into mice. Aliquots of *MmP*ah mRNA-LNPs were prepared and stored at 0.3 g/L. Physicochemical characterization of LNPs demonstrated *MmPah* mRNA encapsulation efficiency of 94%, a particle diameter of 73 nm, and a homogenous size distribution of 0.08 as judged by polydispersity index (PdI). CRE mRNA-LNPs had a particle size of 83 nm, a PdI of 0.04, and an encapsulation efficiency of 96%.

### Mouse phenylketonuria model

A mouse model mimicking phenylketonuria (*BTBR-Pah* ^*enu2*^ */J*, JAX stock number 002232) was obtained from The Jackson Laboratory (Bar Harbor, ME, United States) (Shedlovsky et al., 1993). These mice (abbreviated PKU) were generated by ethylnitrosourea (ENU)-induced random mutagenesis, which resulted in a missense mutation in exon 7. This region is relevant to the active PAH enzyme site. Homozygous *Pah* ^*enu2*^ animals exhibit elevated serum L-Phe levels, are mildly growth restricted and cognitively impaired. The model resembles a phenotype of untreated severe PKU in humans with BH_4_-non-responsive characteristics (van Spronsen et al., 2021). C57BL/6J mice purchased from The Jackson Laboratory (Bar Harbor, ME, United States) were used as negative controls. Male mice were used in all experiments to avoid gender-related variability. Thus, the mRNA-based therapy was tested as a proof-of-concept with a limited set of mice according to the 3R principle applied in animal research.

### Biodistribution

The reporter mouse strain, *B6.Cg-Gt(ROSA)26Sor* ^*tm14(CAG-tdTomato)Hze*^ */J* (JAX stock number 007914), was obtained from The Jackson Laboratory (Madisen et al., 2010). This strain (abbreviated CRE) contains a loxP-flanked STOP cassette preventing transcription of a red fluorescent protein variant (tdTomato). CRE mice express robust tdTomato fluorescence only in the presence of functional CRE protein. CRE-encoding mRNA was incorporated into LNPs (*Cre* mRNA-LNPs) and used to assess the biodistribution of mRNA-LNPs injected IV. Cohorts of 5 PKU mice were injected IV (200 μl, mRNA dose 60 μg) according to the following experimental groups: 1) CRE mice injected with *Cre* mRNA-LNPs, 2) CRE mice injected with NaCl, 3) C57BL/6J mice injected with *Cre* mRNA-LNPs, and 4) C57BL/6J mice injected with NaCl. Mice were euthanized 24 h after injection, and the organs were dissected for *ex vivo* imaging. Livers were subjected to Western blot analysis. Hepatocytes and nonparenchymal liver cells were purified, lysed, and subjected to FACS analysis.

### Parenchymal and nonparenchymal liver cell isolation

Dissected liver tissues were incubated for 30 min at 37°C in dissociation medium composed of Leibovitz-15 medium (Sigma-Aldrich), 45% glucose, 1 g/L DNAse I (Applichem) and collagenase A (Roche, Basel, Switzerland). Digested liver fragments were passed through a nylon cell strainer, and cells free in suspension were collected in 50 ml wash medium/liver and stored at 4°C. Cells were centrifuged at 30 × g for 15 min at 4°C to pellet parenchymal cells, i.e., hepatocytes. Parenchymal cells were washed 3 times with FACS buffer and analyzed by flow cytometry.

Non-parenchymal cells (NPCs) remaining in suspension were collected and centrifuged at 300 × g for 10 min at 4°C. Pelleted cells were resuspended in ice-cold-HBSS, mixed with freshly prepared 30% Histodenz (Sigma-Aldrich), and centrifuged at 1,500 × g for 20 min at 4°C. The NPCs at the Histodenz interface were collected, washed, and suspended in FACS buffer for analysis. The NPCs were phenotyped using dye-conjugated monoclonal antibodies specific for CD45 (clone 30-F11), F4/80 (clone BM8), and CD31 (clone 390) (BD Biosciences, Franklin Lake, NJ, United States). Stained samples were acquired on a multichannel cytometer BD LSR II equipped with FACS Diva software (BD Biosciences; Franklin Lake, NJ, United States) and analyzed using FlowJo 7.6.5 (Becton, Dickinson Company; Ashland, OR, United States).

### Single intravenous injections of *MmPah* mRNA-LNPs

Groups of PKU mice were either: 1) uninjected or 2) injected once IV with a dose of 60 μg of *MmPah* mRNA formulated in LNPs (*MmPah* mRNA-LNPs). Cohorts of *MmPah* mRNA-LNPs injected mice (5 mice/group) were euthanized on days 1, 2, and 4 post injection; 5 untreated control mice were euthanized on day 1. Body weights of PKU mice were determined before injection and at time of euthanasia. Blood was collected 4 days prior to injection to determine pre-treatment metabolite levels; blood and organs were collected at time of euthanasia for further analyses.

### Repeated intravenous injections of *MmPah* mRNA-LNPs

PKU mice were injected IV once every 5 days (5 times in total) with *MmPah* mRNA-LNPs. Uninjected mice served as controls. Body weights were determined before the first injection, throughout the experiment, and at time of euthanasia (day 21). Blood was collected 5 days prior to treatment, 24 h after each injection and at time of euthanasia. Livers and brains were collected for further analyses.

### Phenylalanine and tyrosine quantitation

Phe and Tyr were measured using an automated Biochrom 30 amino acid analyzer (Biochrom Ltd., Cambridge, United Kingdom) according to standardized procedures (Duran, 2008).

### Alanine transaminase and aspartate transaminase quantitation

ALT and AST levels in mouse serum were quantified using an Abbott Alinity ci-serie module (Abbott Laboratories, Chicago, IL, United States). Reagents and standardized protocols were used according to Abbott recommendations.

### Western blot analysis

Tissues and cells were lysed in 1 ml RIPA buffer supplemented with Complete Mini Protease Inhibitor cocktail (Roche). Lysates were diluted and mixed with loading buffer containing ß-mercaptoethanol. Samples were heated for 5 min at 95°C; vortexed, centrifuged at 14.000 rcf at room temperature and 12 μl of the supernatants were transferred to 26-well, 10% Criterion™ TGX™ Precast Midi Protein Gel (Bio-Rad Hercules, CA, United States). Gels were electrophoresed at 150 V for 75 min. Wet transfers were performed at 100 V for 60 min with Mini Trans-Blot (Electrophoretic Transfer Cell/Bio Rad) in 1X transfer buffer. Membranes were blocked with 1x TBS and 5% skim milk in H_2_O for 45 min at room temperature with shaking, then washed and incubated for 1–2 h at room temperature with primary antibodies: polyclonal rabbit anti-PAH (Abcam, Cambridge, United Kingdom), anti-RFP antibody (rabbit polyclonal) [ab62341] or monoclonal mouse anti-ß-actin (Abcam), which served as loading control. Membranes were washed again, incubated with secondary antibodies [IRDye 800CW-conjugated goat anti-rabbit IgG (LI-COR Biosciences, Lincoln, NE, United States) or IRDye 680RD-conjugated goat anti-mouse IgG (LI-COR)] for one hour at room temperature followed by washing. An Odyssey CLX imaging system (LI-COR) was used for quantitation and analysis.

### Histochemical analysis

Formalin-fixed, paraffin embedded liver tissue was cut into 2 μm sections and stained with hematoxylin and eosin.

### Statistical analysis

Statistical analyses were performed with Prism (Graphpad Software, San Diego, CA, United States) using one- and two-way ANOVA, or a two-tailed Student’s *t* test. Data are the means ± SEM. *p* values < 0.05 were considered significant.

## Results

### *MmPah* mRNA transfection *in vitro* results in the production of functional phenylalanine hydroxylase protein

**FIGURE 1:**
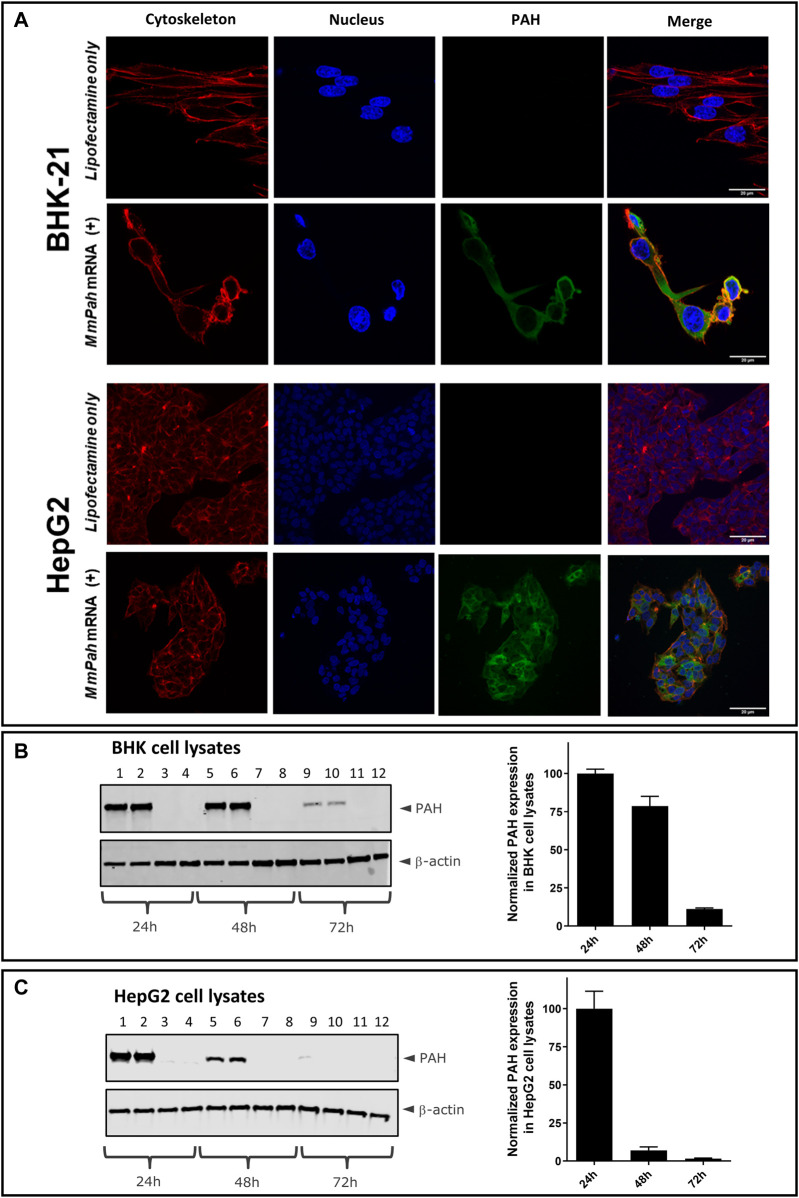
Production and localization of PAH in BHK and HepG2 cells transfected with *MmPah* mRNA. **(A)** BHK-21 and HepG2 cells were transfected with *MmPah* mRNA in Lipofectamine 24 h after plating. Control cells were treated with Lipofectamine only. At 24 h after transfection, PAH protein stained with dye-conjugated anti-PAH antibody was visualized by confocal laser scanning microscopy; actin filaments and cell nuclei were stained with PhalloidinTexasRed and DAPI, respectively. **(B,C)** Western blot analysis of BHK **(B)** and HepG2 cell lysates **(C)** collected at 24, 48, and 72 h after transfection. Lanes 1, 2, 5, 6, 9, 10 correspond to transfection with *MmPah* mRNA-Lipofectamine; lanes 3, 4, 7, 8, 11, 12 correspond to cell lysates after treatment with Lipofectamine only. Data correspond to two independent experiments performed by two investigators with duplicates each (resulting in *n* = 4 in total). Quantitation of Western blots for BHK [**(B)**, right] and HepG2 [**(C)**, right] at different time points after transfection. Shown are PAH expression levels normalized to ß-actin; PAH expression levels at time point 24 h set to 100% for comparison of different experiments.

Two different cell types were transfected with *MmPah* mRNA to evaluate PAH protein production *in vitro*: BHK-21 cells, characterized by the absence of endogenous PAH; and HepG2 cells, which exhibit a hepatocyte-like phenotype. Hepatocytes represent the target cell for LNP-mediated *MmPah* mRNA delivery *in vivo*. Confocal laser scanning microscopy (CLSM) revealed PAH production and intracellular localization 24 h after transfection of either BHK-21 or HepG2 cells (Figure 1A). Western blot analysis of BHK and HepG2 cell lysates collected at different time points after transfection of *MmPah* mRNA complexed in Lipofectamine versus Lipofectamine control showed highest expression at 24 h which decreases subsequently over time (as measured until 72 h *in vitro*) (Figures 1B,C).

**FIGURE 2:**
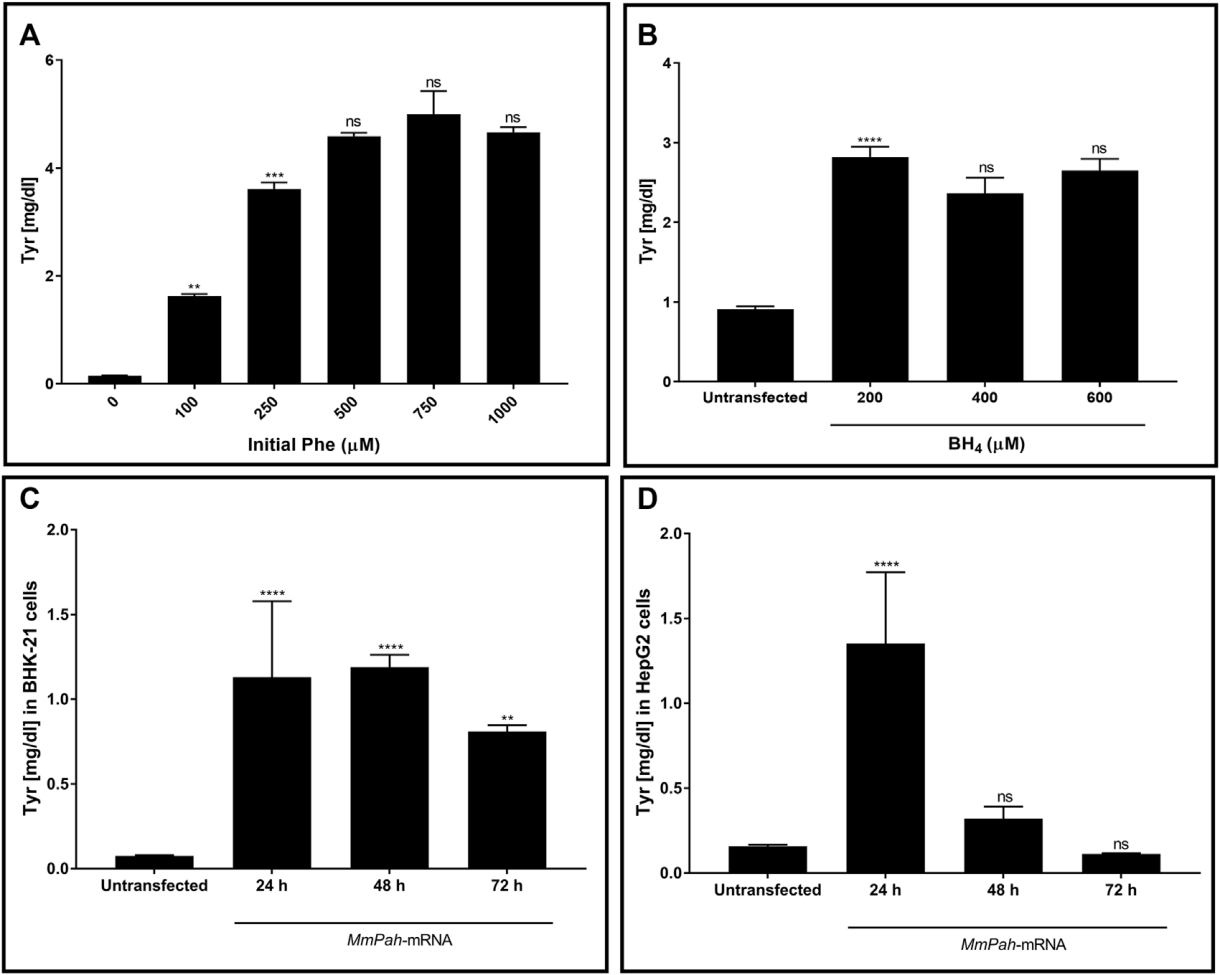
PAH protein synthesized by cells transfected with *MmPah* mRNA is functional. **(A)** Effect of increasing Phe concentrations on PHA enzyme activity was evaluated. Bars represent the amount of Tyr production quantified at the end of the assay period. Data are the means ± SEM obtained in 3 independent experiments. Significantly different from the value obtained at the adjacent lower Phe concentration: no significant differences (ns); **p < 0.01; ***p < 0.001 (one-way ANOVA, Tukey’s multiple comparisons test). **(B)** Effect of increasing BH_4_ concentrations on enzyme activity assessed for Tyr production. Phe concentration was fixed to 500 μM. **(C)** BHK-21 and **(D)** HepG2 cell lysates were transfected with MmPah mRNA or control. Enzymatic activity and Tyr production were assessed at times indicated. Results are the means ± SEM Tyr concentration obtained in 2 independent experiments. Significance tested compared to the untransfected condition: no significant differences (ns); **p < 0.01; ****p < 0.0001 (one-way ANOVA, Tukey’s multiple comparisons test).

The enzymatic activity of PAH produced *in vitro* was tested. Initially, changes in activity were assessed after incubating the protein with increasing substrate (Phe) concentrations. Enzymatic activity, assessed *via* Tyr production, increased with increasing Phe (Figure 2A; Supplementary Figure S1A). No further increase in Tyr production occurred at Phe concentrations > 250 μM, demonstrating substrate saturation of the enzyme. The effect of increasing BH_4_ concentrations on enzyme activity was also tested but was found to have no apparent impact (Figure 2B; Supplementary Figure S1B). In correlation with WB results, the enzymatic activity in both BHK-21 and HepG2 cells peaked at 24 h after transfection then decreased markedly over time (Figures 2C,D; Supplementary Figures S1C,D). Taken together, these results demonstrate the correct localization and functionality of the PAH protein after transfection of *MmPah* mRNA *in vitro*; these results provide the rationale for evaluating the efficacy of *MmPah* mRNA expression in a PKU disease model.

### Biodistribution of mRNA-LNPs *in vivo*

**FIGURE 3:**
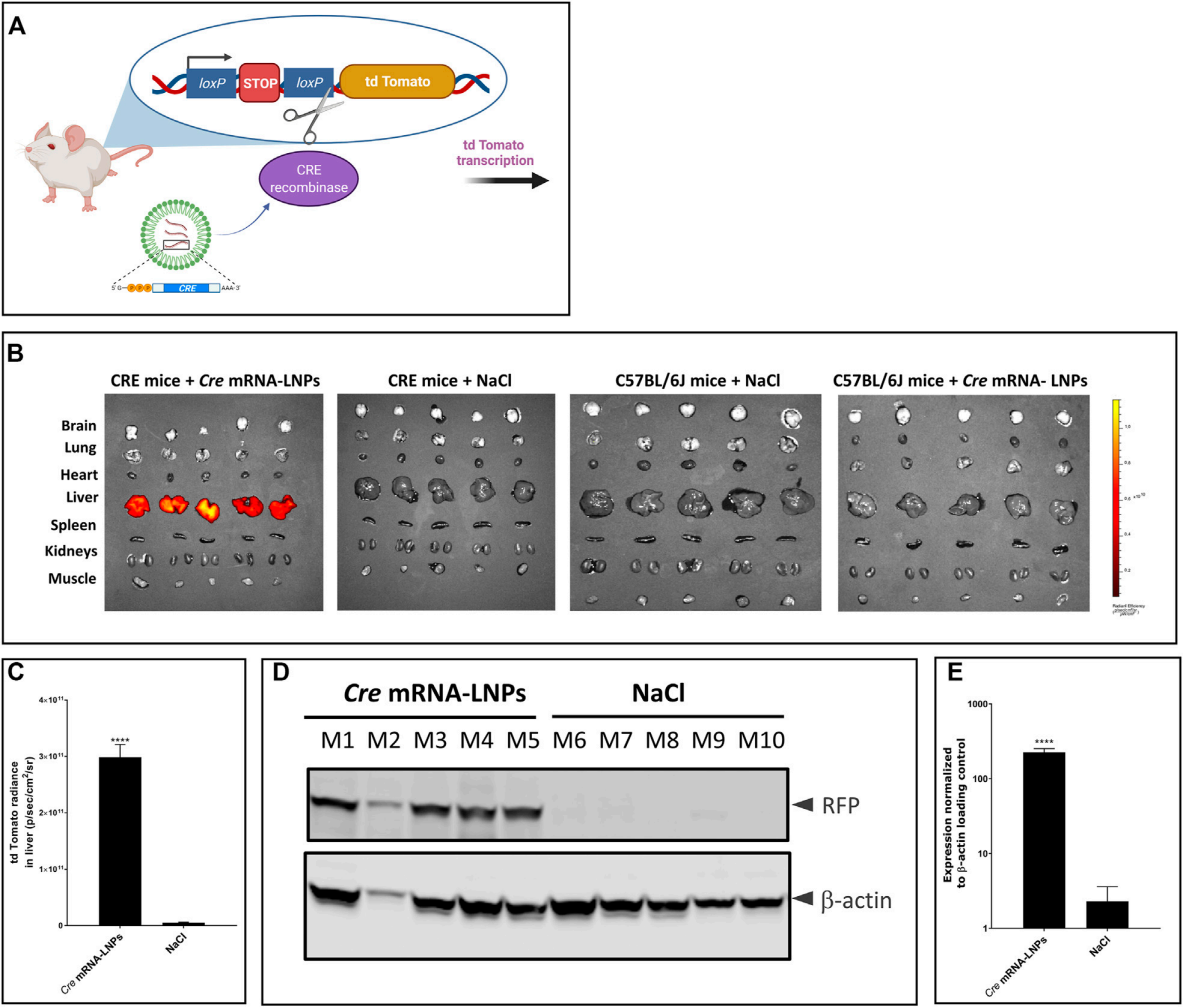
*Cre* mRNA-LNPs injected IV into CRE reporter mice elicits reporter gene activation in the liver. **(A)** Schematic representation of the reporter mouse strain, B6.Cg-Gt(ROSA)26Sor^tm14(CAG-tdTomato)Hze^/J (CRE-mouse). **(B)** CRE mice were injected IV and euthanized 24 h later. *Ex vivo* imaging of tissues are shown for: Group 1, CRE mice injected with *Cre* mRNA-LNPs; Group 2, CRE mice injected with NaCl; Group 3, C57BL/6J mice injected with *Cre* mRNA-LNPs; Group 4, C57BL/6J mice injected with NaCl. **(C)** Quantification of tdTomato fluorescent signal obtained for livers after *ex vivo* imaging. **(D)** Western blot analysis of tdTomato protein (visualized by anti-RFP antibody; RFP, Red Fluorescent Protein) in CRE mouse livers normalized to β-actin loading control. **(E)** Quantitation of tdTomato protein bands normalized to β-actin loading control. Results are the means ± SEM for groups of mice (*n* = 5) injected with *Cre* mRNA-LNPs or saline. Statistically significant difference to the saline control: ****p < 0.0001 (two-tailed Student’s *t*-test).

The reporter mouse strain, *B6.Cg-Gt(ROSA)26Sor* ^*tm14(CAG-tdTomato)Hze*^ */J* (CRE-mice), was used to analyse the biodistribution of LNP-formulated *Cre* mRNA after single IV injection. This CRE reporter strain contains a loxP-flanked STOP cassette that prevents transcription of a red fluorescent protein variant (tdTomato). CRE animals express robust tdTomato fluorescence only in the presence of functional CRE recombinase as depicted in Figure 3A. *Cre*-encoding mRNA was formulated in LNPs (*Cre* mRNA-LNPs) and injected IV into CRE mice. Twenty-four hours later, mice were euthanized, and organs were dissected and imaged *ex vivo* (Figure 3B). Three control groups were evaluated in parallel. Group 2 consists of CRE-mice injected with NaCl; group 3 and group 4 correspond to wild type C57BL/6J mice injected with NaCl 0.9% and *Cre* mRNA-LNPs, respectively. Control groups 2–4 confirmed the absence of endogenous fluorescence and a tdTomato signal in the absence of *Cre* mRNA expression (Figure 3B). In contrast, Group 1 showed an intense tdTomato signal due to the release of *Cre* mRNA from LNPs and expression of functional CRE recombinase in the targeted liver tissue (Figures 3B,C). Livers of injected mice were dissected, lysed, and analyzed by Western blot. *Cre* mRNA-LNP injected CRE mice (mouse M1–M5) showed a strong tdTomato signal detected in their livers with anti-RFP antibody: CRE mice injected with NaCl (mouse M6–M10), on the other hand, did not show any evidence of reporter protein production (Figures 3D,E).

*Cre* mRNA formulated in LNPs resulted in CRE protein production especially in the livers after single IV injection. The protein was functional, capable of excising the STOP cassette and facilitating tdTomato reporter protein synthesis. Intense signals in the livers of group 1 were detected *ex vivo* 24 h after *Cre* mRNA-LNP injection. Moreover, translation of Cre mRNA was detected in CD45^+^ nonparenchymal liver cells: CD45^+^F4/80^+^ Kupffer cells, and CD45^+^CD31^+^endothelial cells (Supplementary Figures S2A,B).

### Single IV *MmPah* mRNA-LNPs injections decrease phenylalanine levels in phenylketonuria mice

**FIGURE 4:**
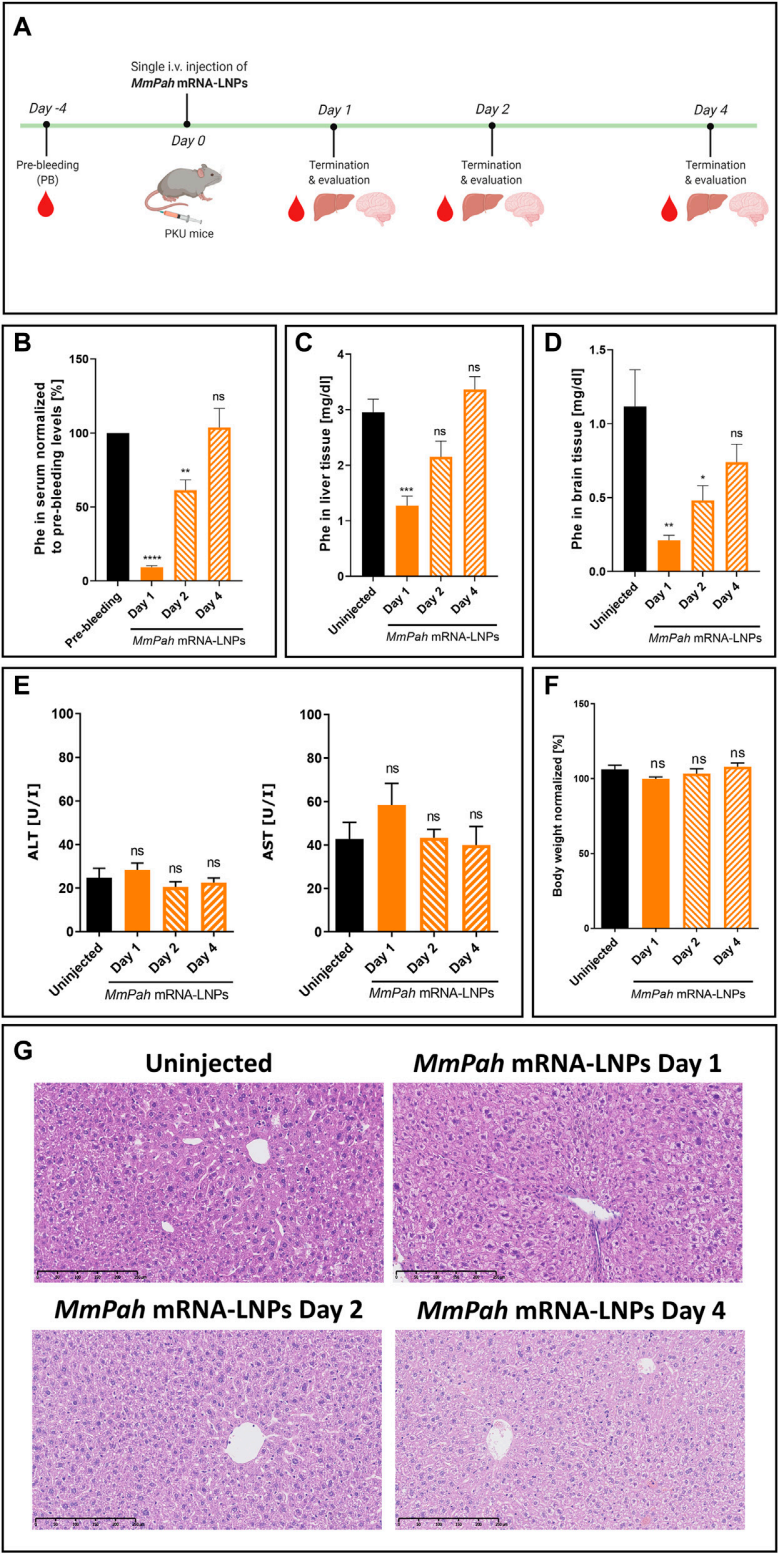
Single intravenous *MmPah* mRNA-LNPs injections reduce Phe levels in serum, livers, and brains of PKU mice. **(A)** Experiment schedule. Blood was collected on day 4 before treatment (PB, pre-bleeding). Blood, livers, and brains were collected on days 1, 2, and 4 after single IV injection. **(B)** Normalized Phe levels in PKU mouse serum at the times indicated (5 mice/group). Absolute Phe levels in PKU mouse **(C)** livers and **(D)** brains on the day indicated post-injection. **(E)** Alanine transaminase (ALT) and aspartate transaminase (AST) in PKU mouse serum at the times indicated post-injection. **(F)** Normalized body weights on the day indicated post-injection. **(G)** Livers were dissected from uninjected or *MmPah* mRNA-LNPs injected PKU mice on the day indicated, embedded in paraffin, sectioned, and stained with H&E. Results are the means ± SEM of each animal cohort (*n* = 5). Significantly different from uninjected group or pre-bleeding control: no significant difference (ns); *p < 0.05; **p < 0.01; ***p < 0.001; ****p < 0.0001 (one-way ANOVA, Dunnett’s multiple comparisons test).

*MmPah* mRNA formulated in LNPs was injected IV into PKU mice. Animals fed a normal diet with no Phe or Tyr restrictions, exhibited high serum Phe levels prior to therapy. After a single injection of *MmPah* mRNA-LNPs IV, mice were euthanized on days 1, 2, and 4, and the therapeutic effects of *MmPah* mRNA were evaluated and compared to non-treated PKU mice (Figure 4A). Serum was collected 4 days prior to treatment to evaluate baseline metabolite levels. Treatment resulted in a 10-fold decrease in Phe serum levels 24 h after injection; an approximate 50% reduction was still apparent 48 h after *MmPah* mRNA-LNPs injection (Figure 4B). At later time points, serum Phe levels increased to pre-treatment values. Phe levels evaluated in livers and brains correlated with those obtained in serum, i.e., decreasing early then returning to pretreatment levels (Figures 4C,D). Serum levels of ALT and AST (markers of liver damage), cytokines, and chemokines did not show significant differences between treated and control mice (Figure 4E; Supplementary Figure S3B). Body weight was not affected by treatment with *MmPah* mRNA-LNPs and increased until day 4 (Figure 4F; Supplementary Figure S3A). Histologic examination of paraffin embedded hematoxylin and eosin (H&E)-stained liver sections confirmed the absence of adverse *MmPah* mRNA-LNPs-dependent effects on the liver. Normal liver architecture was preserved after treatment; no infiltrating immune cells were observed at the time points analyzed (Figure 4G).

### Repeated IV *MmPah* mRNA-LNPs injections elicit sustained therapeutic effects

**FIGURE 5:**
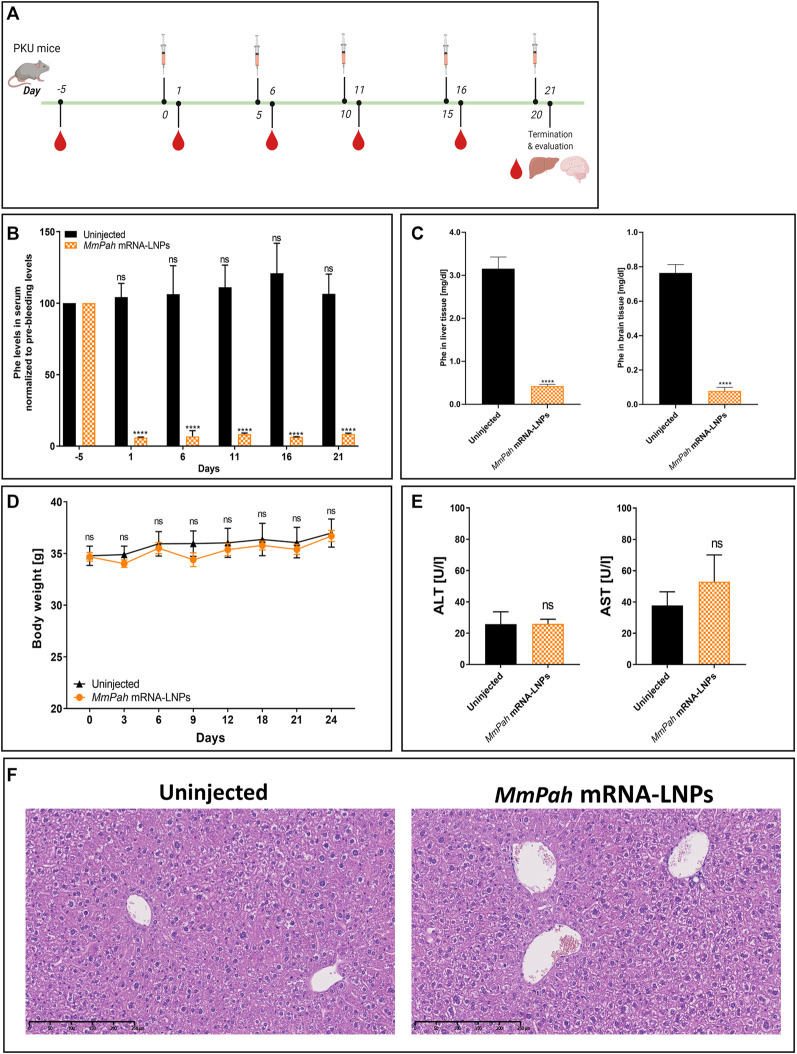
Repeated intravenous injections of *MmPah* mRNA-LNPs IV reduce Phe levels in serum, livers, and brains of PKU mice. **(A)** Experiment schedule. Blood was collected on day -5 before treatment (PB, pre-bleeding), at intermittent times 24 h after each injection, i.e., day 1; day 6; day 11; day 16), and on termination day 21. Uninjected PKU mice were used as control cohort. LNP-formulated therapeutic *MmPah* mRNA was injected IV a total of 5 times once every 5 days (days 0, 5, 10, 15, and 20) in the experimental cohort (*MmPah* mRNA-LNPs). **(B)** Normalized serum Phe levels on the days indicated. **(C)** Comparison of absolute Phe levels in livers and brains of control, untreated and *MmPah* mRNA-LNPs injected PKU mice. **(D)** Body weights determined on the days indicated. **(E)** Levels of ALT and AST were evaluated in PKU mouse serum on day 21. **(F)** Paraffin-embedded, H&E-stained liver sections derived from *MmPah* mRNA-LNPs treated or untreated mice. Results are the means ± SEM for groups of 5 mice. Significantly different from the untreated control group: no significant differences (ns); ****p < 0.0001 [two-way ANOVA, Sidak’s multiple comparisons test **(A,B)**; two-tailed Student’s *t*-test **(C,D)**.

An experimental approach was designed to administer *MmPah* mRNA-LNPs to PKU mice repeatedly. *MmPah* mRNA-LNPs were injected IV once every 5 days for 5 times for a total experimental period of 21 days according to the schedule depicted in Figure 5A. Serum was collected 5 days before start of treatment, 24 h after each injection, and on termination day 21. A significant reduction in Phe levels was observed at 24 h after each injection, reaching physiological values (Figure 5B). Similarly, Phe levels were significantly decreased in liver and brain tissues of *MmPah* mRNA LNP-treated versus untreated PKU mice (Figure 5C). Repeated administration of *MmPah* mRNA-LNPs was very well tolerated. There was no effect on body weight (Figure 5D), liver enzymes (ALT and AST) levels (Figure 5E), or cytokine and chemokine levels (Supplementary Figure S4) in PKU mouse serum. Histologic examination of paraffin-embedded sections of livers dissected on day 21 after repeated *MmPah* mRNA-LNPs injection revealed no changes in liver architecture compared to untreated PKU mice (Figure 5F). Taken together, these results demonstrate that *MmPah* mRNA-based therapy targeted to the liver expressed functional PAH protein, which is well tolerated and effective in reducing the accumulation of Phe in serum, livers, and brains of PKU mice.

## Discussion

The lack of PAH enzyme function results in the accumulation of Phe in blood and tissues of PKU patients. Untreated patients often develop intellectual, behavioral, and psychiatric problems. Early diagnosis in combination with a life-long Phe-restricted diet prevents the severe effects of disease. Although some neuropsychological problems have been reported in treated PKU patients, the burden of adhering to a strict life-long diet constitutes one of the main reasons for developing new treatment options. Patients having access to a novel biopharmaceutical formulation that decreases the accumulation of toxic metabolites in a timely and accurate fashion in combination with the possibility to ease the strict diet would benefit most from the development of such alternate treatments. mRNA-based therapies comprise a potent tool to meet the needs of different indications ranging from infectious disease vaccines to protein replacement therapies. Indeed, genetic approaches to treat diseases associated with the absence or dysfunction of essential proteins have been heavily investigated in recent years. Potential unwanted off-target effects associated with adenovirus vectors and gene-editing tools such as CRISPR/Cas9, undermine the potential use of these approaches to treat human disease. mRNA-based approaches, on the other hand, offer a means of confronting these challenges using a clinically proven technology that does not require changes in the genetic information encoded by the affected cell(s) to generate a desired therapeutic effect. Importantly, during metabolic crises, or during events when not strictly adhering to the diet, strong increases in Phe levels occur inducing severe neurological effects. mRNA-based therapy could therefore be applied in emergency use, as PAH protein is quickly synthesized consequently returning Phe to safe levels, especially in the brain.

Baby hamster kidney cells (BHK-21) and human hepatocyte-like cells (HepG2) transfected with murine *MmPah* mRNA exhibited robust intracellular PAH production evaluated by Western blot and CLSM. *MmPah* mRNA encoded a functional protein (PAH) that effectively degraded Phe to Tyr in an enzyme activity assay. Notably, both Phe- and BH_4_-dependent PAH enzyme kinetics clearly showed data that seem to fit with sigmoidal equation like Hill kinetic model (Figures 2A,B). Previous reports for human PAH stated that enzyme kinetics adjust to Hill kinetic model (Gersting et al., 2010; Staudigl et al., 2011). Findings demonstrating the saturation of PAH activity at Phe concentrations > 250 μM correlate with previous reports by other investigators (Pey and Martinez, 2005; Staudigl et al., 2011). PAH activity was observed at all tested BH_4_ concentrations. The response of PAH to BH_4_ is relevant since the cofactor stabilizes the enzyme and naturally affects its function (Flydal and Martinez, 2013). Remarkably, fluctuations in PAH activity in response to Phe concentration resemble physiologic conditions that occur *in situ* where Phe levels change mainly in response to food intake. These results support an mRNA-based therapeutic approach to generating a functional enzyme that exhibits characteristics that are very similar to those of native human PAH.

Biodistribution studies conducted in reporter animals showed expression of Cre protein after mRNA-LNP injection primarily localizing in liver with little or no expression apparent in other organs. Of note, hepatocytes constitute the principal site of our therapeutic approach to produce functional PAH, although we found, that nonparenchymal liver cells (mainly Kupffer cells and endothelial cells) also produced CRE protein, as a consequence of their strong phagocytic activity and ability to internalize mRNA-LNPs. Like many other nanocarriers, LNPs’ bioavailability is often reduced by the clearance in liver tissue. This is a limitation for the effective systemic delivery of mRNA. Thus, this phenomenon remains as a complex barrier to overcome for successful hepatocyte delivery. In this context, achieving near 30% of tdTomato^+^ hepatocytes by our LNP formulation could be considered as a successful delivery of the mRNA construct.

Therapeutically active *MmPah* mRNA was formulated in LNPs and injected into PAH-deficient mice, which represent a widely used model of severe PKU in humans. This mouse model is characterized by the complete absence of PAH activity, thereby accurately representing human disease. PKU mice normally exhibit elevated levels of L-Phe in serum and cognitive impairment. Phe levels in serum and organs served as direct and effective parameters to assess therapeutic potential. A single fixed 60 μg dose of *MmPah* mRNA-LNPs injected IV significantly reduced Phe levels in serum, livers, and most importantly in brains of PKU mice on days 1 and 2 after injection. The therapeutic effect vanished by day 4, however, due to the relatively short half-life of the PAH protein. *MmPah* mRNA generated a functional PAH enzyme *in vivo* that was capable of metabolizing Phe. Treatment was well tolerated, highlighting the potential use of mRNA-based therapy in a chronic setting.

PKU mice were injected repeatedly with *MmPah* mRNA to mimic chronic short-term treatment of PKU patients. Mice were injected a total of 5 times at 5-day intervals over the course of a 21-day period. A similar schedule was investigated in an experimental mouse model of hereditary spastic paraplegia type 5 (Hauser et al., 2019) and in a Tyrosinemia mouse model (Cacicedo et al., 2022) The repeated intravenous injection of therapeutic mRNA formulated in LNPs induced protein expression in the liver and, consequently, a significant reduction in the concentration of neurotoxic metabolites in serum, liver, and brain of treated, relative to nontreated, animals.

The repeated administration of *MmPah* mRNA-LNPs was well-tolerated, having no apparent effects on body weight, liver function (ALT and AST levels, markers of liver damage, were normal), or the production of pro-inflammatory cytokines and chemokines. Importantly, Phe levels were low 24 h after each injection and remained low after the fifth injection evidencing the stability of the therapeutic effect upon repeated administration. Moreover, Phe values in the brain were reduced significantly at termination of the repeated *MmPah* mRNA-LNPs injection regimen evidencing the potential effectiveness of therapy and treatment of the most affected organ in PKU patients over a long period.

Notably, the therapeutic effects of *MmPah* mRNA-LNPs were obtained without supplementation with other drug products such as the BH_4_ co-factor analog, sapropterin dihydrochloride. Remarkedly, a dose as low as 60 μg (equivalent to 1.63–1.82 mg/kg/mouse/injection) administered periodically over a 21-day period sustained the body weight of PKU-deficient mice on a normal diet, and drastically reduced levels of Phe in serum, liver, and brain. These findings provide the potential basis for the clinical application of mRNA technology to treat human PKU patients. Although the current mRNA-based formulation could be potentially applied as an emergency-use option, or as a supplement to dietary treatment, its administration could be extended as a chronic therapeutic treatment replacing life-long diet. A short-lasting therapeutic effect could be prolonged by optimizing mRNA and/or protein half-life, thereby expanding the therapeutic window between repeated administrations.

During preparation of this manuscript, *in vivo* studies in the Pah^enu2^ mouse model using mRNA-LNPs encoding human PAH were reported (Perez-Garcia et al., 2022). Here, our LNP-formulated mouse Pah mRNA proofed to be therapeutically active at lower doses (1.63–1.82 mg/kg in comparison to 3–10 mg/kg in the Perez-Garcia et al., 2022 study) in lowering Phe in PKU mouse serum after each of the five injections, and most importantly to reduce Phe in brain, the most affected organ in PKU patients.

## Data Availability

The original contributions presented in the study are included in the article/Supplementary Material, further inquiries can be directed to the corresponding author.
