# Supplementary material for: Phenylalanine hydroxylase mRNA rescues the phenylketonuria phenotype in mice
Source: Front Bioeng Biotechnol. 2022 Oct 7;10:993298. doi: 10.3389/fbioe.2022.993298 (PMC9585315; doi:10.3389/fbioe.2022.993298)
Supplement: Supplementary file 1 [file DataSheet1.docx]

Supplementary Material

# Supplementary Figures


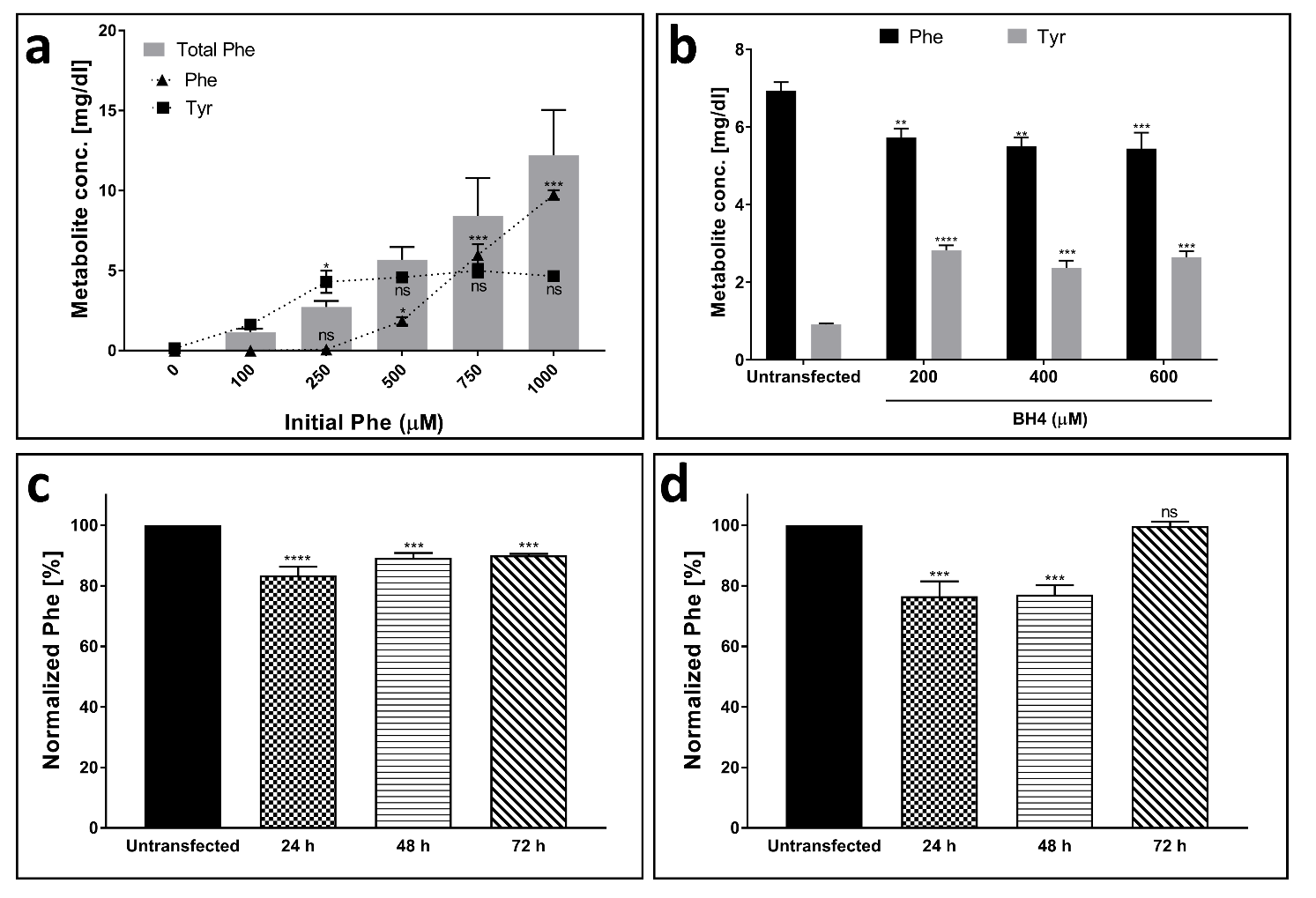


**Supplemental Figure 1. *In vitro* produced *MmPah* protein proofs enzymatic activity by metabolizing Phe in Tyr.**

**(a)** *MmPah* enzymatic activity was evaluated at different Phe concentrations to test the response of the enzyme to its substrate. Bars express the amount of Phe at time zero. Triangle and square symbols represent Phe and Tyr concentrations measured after enzymatic activity assay, respectively. A substrate-mediated saturation of the enzymatic activity was observed. Results were analyzed as mean ± SEM from 3 independent experiments; two-way ANOVA, Tukey's multiple comparisons test. Significantly different from the previous value: no significant differences (ns), *P <0.05, **P <0.01, ***P <0.001, ****P <0.0001). **(b)** Different BH_4_ concentrations were tested in the enzymatic assay, with no apparent differences regarding Phe degradation by the PAH enzyme. Results were analyzed as mean ± SEM from 3 independent experiments; two-way ANOVA, Dunnett's multiple comparisons test. Significantly different from untransfected control: no significant differences (ns); *P <0.05; **P <0.01; ***P <0.001; ****P <0.0001). **(c)** BHK-21 and **(d)** HepG2 cells were transfected with *MmPah* mRNA. After 24h, 48h or 72h enzymatic activity was measured. Phe values, normalized to initial Phe concentrations, are depicted. Results were analyzed as mean ± SEM from 2 independent experiments; one-way ANOVA, Tukey's multiple comparisons test. Significantly different from untransfected samples: no significant differences (ns); *P <0.05; **P <0.01; ***P <0.001; ****P <0.0001.


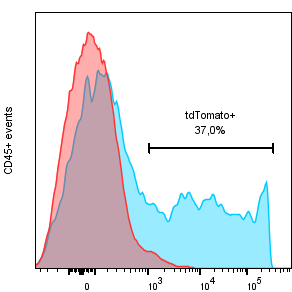

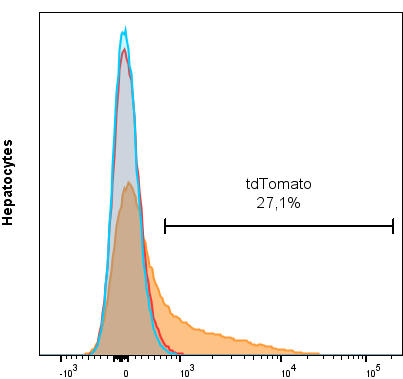

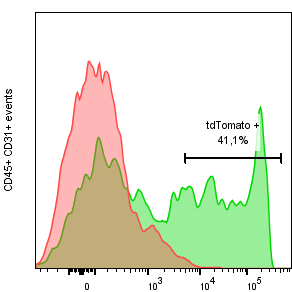

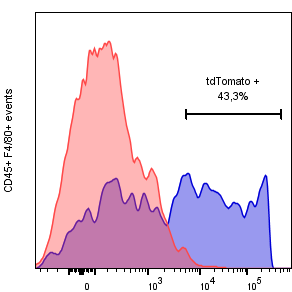


**b**

**a**

**Supplemental Figure 2. Cell-type specific CRE protein expression in a biodistribution study in PKU mice after single IV injection.**

**(a)** Liver non-parenchymal cells (NPCs) were isolated and analyzed via flow cytometry. Fluorescent signal from tdTomato was analyzed in Kupffer cells (CD45^+^ F4/80^+^) and endothelial cells (CD45^+^ CD31^+^). Only those animals injected with *Cre* mRNA-LNPs showed presence of tdTomato fluorescence (green color for KCs and violet for ECs in the histograms). **(b)** Flow cytometry analysis of hepatocytes showed tdTomato fluorescence in CRE mice injected with *Cre* mRNA-LNPs.

**a**

**b**

**Supplemental Figure 3. Body weights of PKU mice and cytokine and chemokine levels in PKU mouse serum after single intravenous injection of *MmPah* mRNA-LNPs.**

**(a)**  Body weights of PKU mice measured before start of injection (PB = pre-bleeding time point Day -4), on day of single IV injection (Day 0) and on indicated time points after injection (up to Day 4). PKU mice were injected with therapeutically active *MmPah* mRNA-LNPs or left uninjected (n=5 mice per group). **(b)** Cytokine and chemokine levels in PKU and wildtype mouse serum measured at termination. PKU mice were treated with *MmPah* mRNA-LNPs in single IV injection as indicated. Wildtype animals were not injected. Results were analyzed as mean ± SEM; one-way or two-way ANOVA were performed with Dunnett's multiple comparisons test. Significantly different from uninjected or PB control: no significant differences (ns), p>0,05.

**Supplemental Figure 4. Cytokine and chemokine levels in PKU mouse serum after repeated intravenous injection of *MmPah* mRNA-LNPs.**

Cytokine and chemokine levels in PKU mouse serum measured at termination on day 21 after repeated IV injections of *MmPah* mRNA-LNPs in comparison to uninjected PKU mice. Results were analyzed as mean ± SEM; Two-tailed Student´s t-test. Significantly different from uninjected PKU mice: no significant differences (ns); **** P <0.0001.
